# Congruent Strain Specific Intestinal Persistence of *Lactobacillus plantarum* in an Intestine-Mimicking *In Vitro* System and in Human Volunteers

**DOI:** 10.1371/journal.pone.0044588

**Published:** 2012-09-06

**Authors:** Hermien van Bokhorst-van de Veen, Iris van Swam, Michiel Wels, Peter A. Bron, Michiel Kleerebezem

**Affiliations:** 1 TI Food & Nutrition, Wageningen, The Netherlands; 2 NIZO food research, Ede, The Netherlands; 3 Laboratory of Microbiology, Wageningen University and Research Centre, Wageningen, The Netherlands; 4 Centre for Molecular and Biomolecular Informatics (CMBI 260), Radboud University Medical Centre, Nijmegen, The Netherlands; 5 Kluyver Centre for Genomics of Industrial Fermentation, Delft, The Netherlands; 6 Host-Microbe Interactomics, Wageningen University and Research Centre, Wageningen, The Netherlands; Loyola University Medical Center, United States of America

## Abstract

**Background:**

An important trait of probiotics is their capability to reach their intestinal target sites alive to optimally exert their beneficial effects. Assessment of this trait in intestine-mimicking *in vitro* model systems has revealed differential survival of individual strains of a species. However, data on the *in situ* persistence characteristics of individual or mixtures of strains of the same species in the gastrointestinal tract of healthy human volunteers have not been reported to date.

**Methodology/Principal Findings:**

The GI-tract survival of individual *L. plantarum* strains was determined using an intestine mimicking model system, revealing substantial inter-strain differences. The obtained data were correlated to genomic diversity of the strains using comparative genome hybridization (CGH) datasets, but this approach failed to discover specific genetic loci that explain the observed differences between the strains. Moreover, we developed a next-generation sequencing-based method that targets a variable intergenic region, and employed this method to assess the *in vivo* GI-tract persistence of different *L. plantarum* strains when administered in mixtures to healthy human volunteers. Remarkable consistency of the strain-specific persistence curves were observed between individual volunteers, which also correlated significantly with the GI-tract survival predicted on basis of the *in vitro* assay.

**Conclusion:**

The survival of individual *L. plantarum* strains in the GI-tract could not be correlated to the absence or presence of specific genes compared to the reference strain *L. plantarum* WCFS1. Nevertheless, *in vivo* persistence analysis in the human GI-tract confirmed the strain-specific persistence, which appeared to be remarkably similar in different healthy volunteers. Moreover, the relative strain-specific persistence *in vivo* appeared to be accurately and significantly predicted by their relative survival in the intestine-mimicking *in vitro* assay, supporting the use of this assay for screening of strain-specific GI persistence.

## Introduction

Probiotics are defined as ‘live microorganisms which, when administered in adequate amounts, confer a health benefit on the host’ [1]. The most widely applied probiotics belong to the genera *Lactobacillus* and *Bifidobacterium* [2], [3]. To be able to exert their beneficial effects in the intestine, it is a prerequisite for probiotic cultures to counteract the stressful conditions encountered during production, shelf life, and exposure to the harsh conditions of the (upper) digestive tract [4], [5].

A straightforward strategy that is typically applied for the selection of robust probiotic strains is to subject these bacteria to a series of conditions that mimic the gastrointestinal (GI)-tract *in vitro*, including survival at low pH (resembling the stomach) and/or upon exposure to bile salts and digestive enzymes (resembling the duodenum) [6]–[8]. A diverse range of lactobacilli and bifidobacteria have also been tested in more sophisticated GI-tract simulators, e.g. the TNO Intestinal Models (TIM-1 and TIM-2) [9], [10], the Simulator of Human Intestinal Microbial Ecosystem (SHIME) [11], and the Dynamic Gastric Model (DGM) [12]. Although physicochemical properties and/or microbial interactions of the strains of interest can be investigated in these models, they lack the interactions of the bacteria with host cells such as epithelial and immune cells.

Besides the *in vitro* work discussed above, a limited number of *in vivo* studies have assessed the GI survival and persistence of candidate probiotic strains. For example, 7% of the single administered *L. plantarum* NCIMB8826 reached the ileum alive, while of *L. fermentum* KLD and *Lactococcus lactis* MG1363 only 0.5 and 1.0% of the consumed bacteria could be recovered, respectively [13]. In addition, distinct persistence and survival characteristics of *L. gasseri* [14], *L. reuteri* [15], and *L. plantarum* [15] mixed with other species were reported. Moreover, several studies using 3 strains of *L. reuteri* illustrated the wide-range of GI persistence characteristics of these strains, which ranged from detection on 14 to 49 days following consumption by volunteers [16]–[18]. These studies indicate that strains of the same species may display considerable variation in GI-tract persistence. However, this information is only available for very few species, and is restricted to only few strains of these species.

*L. plantarum* is encountered in a variety of artisanal and industrial fermentations, ranging from vegetables to milk and meat [19]. Next to this dietary abundance, *L. plantarum* is frequently encountered as a natural inhabitant of the GI-tract of several mammals, including humans [20], and specific strains are commercially exploited as probiotics [21]. A single colony isolate of *L. plantarum* NCIMB8826, designated *L. plantarum* WCFS1, was the first *Lactobacillus* strain of which the full genome sequence was reported [22]. An *in vitro* GI-tract assay combined with transcriptome-trait matching, followed by mutagenesis approaches [23], established a role of an AraC-family transcription regulator (*lp_1669*), a penicillin-binding protein (Pbp2A), and a Na^+^/H^+^ antiporter (NapA3) in survival under intestinal conditions [7]. Furthermore, specific stress responses in *L. plantarum* have been deciphered [24]–[27], including GI-tract relevant conditions like bile exposure [27], [28]. Finally, studies also have addressed the transcriptional response to specific GI conditions in mice [29], [30] and humans [31].

Here we present the different survival capacities of a set of *L. plantarum* strains in an *in vitro* assay that mimicks the physicochemical conditions encountered during the initial stages of passage through the human GI tract. To validate these findings to the real-life situation, a next-generation sequencing-based method was developed that is able to discriminate individual strains based on a variable intergenic region. This method was employed to quantitatively follow mixtures of *L. plantarum* strains during digestive tract transit in healthy human volunteers, allowing the determination of the competitive population dynamics persistence of 21 *L. plantarum* strains *in vivo*. This approach revealed that strain-specific GI persistence profiles appeared highly stable across volunteers. Moreover, quantitative ranking of *in vivo* human GI-tract persistence levels of the individual strains was significantly correlated to the ranking obtained for the *in vitro* GI-tract survival assay, providing qualitative predictive value to the *in vitro* method used.

## Materials and Methods

### *In vitro* GI-tract Assay

All strains used in this study are listed in Table S1. Strains were grown in 2× chemically defined medium [32] at 37°C. Prior to exposure to the GI-tract assay, the strains were washed in prewarmed PBS at 37°C. The GI-tract assays were performed as described previously for *L. plantarum* WCFS1 [7]. Briefly, gastric juice (GJ) containing freshly added pepsin and lipase was added to the cultures and the samples were incubated at 37°C while rotating at 10 rpm. GJ at a pH of 2.5 was used for cells harvested from logarithmic phase [optical density at 600 nm (OD_600_) = 1.0 as measured photospectroscopically (Ultraspec 2000, Pharmacia Biotech, Cambridge, UK)] and pH 2.4 for stationary phase *L. plantarum* cells (harvested 25 h after inoculation). After 60 min incubation in GJ, the samples were pH-neutralized and pancreatic juice (PJ) containing pancreatin and bile salts was added, followed by incubation for another 60 min. Samples were taken prior to incubation, and after GJ- and PJ-incubation to determine relative survival rates on basis of colony forming units (CFUs) by spot plating of serial dilutions followed by incubation at 30°C for 2 days.

### Human Trial

The study protocol was approved by the Medical Ethical Committee of Wageningen University, registered under number NL29812.081.09, and the study was conducted according to the principles of the Declaration of Helsinki. Volunteers were aged between 18 and 65 years, had no known health problems, consumed no commercially available probiotic products during the month prior to first fecal sample donation, and had a routine defecation frequency of approximately once per day. Participants were asked to maintain their normal diet, whilst not consuming any commercial probiotic products. Exclusion criteria were defined as digestive tract or organ complaints, any symptoms that are likely to be related to a digestive tract disease, intake of antibiotics during the 3 months prior to the experiment, intake of antacids, and pregnancy. Ten healthy volunteers participated in the study, which all signed a written informed consent form and were informed that they could withdraw from the study at any time without providing a reason.

*L. plantarum s*trains were isolated from highly variable habitats (Table S1). Bacterial preparations containing 10 *L. plantarum* strains (Table 1) mixed in equal amounts, based on culture optical density at 600 nm (OD_600_), were prepared essentially as described previously [33]. Briefly, *L. plantarum* strains were cultured at 37°C in MRS (Difco, West Molesey, United Kingdom), washed with peptone-physiologic salt [0.1% (w/v) peptone and 0.85% (w/v) sodium chloride], and mixed in equal amounts [according to their OD_600_ as measured photospectroscopically (Ultraspec 2000, Pharmacia Biotech, Cambridge, UK)]. Cells were collected by centrifugation at 4000×*g* for 10 min at room temperature and pellets were dissolved in 20% (w/v) maltodextrin, 2% (w/v) glucose solution prior to consumption. Each portion contained approximately 10^11^ CFU. Four mixtures were prepared in which a 10-fold dilution range of strain WCFS1 was included in a standard mixture of 9 other strains (ATCC14197, NCTH19-2, CIP104450, CIP104440, KOG18, ATCC8014, LP85-2, 299v, and NC8). Fecal samples were collected on two different days prior to the intake of the bacterial preparation, and subsequently on the day the volunteers received the bacterial preparation (day 0) and daily during the 10 subsequent days, as well as after 14 and 21 days. Fecal samples obtained were stored at −20°C until DNA isolation (see below). Moreover, to detect *L. plantarum* viability, the fecal samples collected from volunteers 1, 4, and 5 on day 1, 2, 3, 5 and 7 were mixed with glycerol [final concentration of approximately 20% (v/v)] and stored at −80°C prior to plating of serial dilutions. To this end, approximately 2 g feces in glycerol were mixed with 1 ml reduced physiological salt [0.1% (w/v) peptone, 0.05% (w/v) cysteine hydrochloride and 0.8% (w/v) sodium chloride; RPS], serial diluted, plated on MRS agar plates containing 50 µg/ml streptomycin and 10 µg/ml tetracycline, to which (most, if not all) *L. plantarum* strains are naturally resistant, and incubated at 37°C. From subject 2, the plates appeared to contain no or hardly any colonies with the typical *L. plantarum* colony-phenotype and these samples were therefore excluded in the analysis. Colonies of the other 2 subjects were collectively recovered from the plates containing a high density of single colonies by the addition of 2 ml RPS followed by gentle scraping using a spatula. After washing with RPS, these suspensions were stored at −20°C prior to DNA isolation (see below).

**Table 1:** Combinations of 10 strains consumed as mixtures by the 10 volunteers (subjects).

Subject	1 to 5	6	7	8	9	10
**Strain** a	**WCFS1**	**WCFS1**	**WCFS1**	**WCFS1**	**WCFS1**	**WCFS1**
	ATCC14917	Lp95	LD3	LD3	ATCC14917	Lp95
	**NCTH19-2**	**NCTH19-2**	**NCTH19-2**	**NCTH19-2**	**NCTH19-2**	**NCTH19-2**
	CIP104450	CIP104450	CIP104450	Q2	Q2	Q2
	CIP104440	H14	CIP104441	CIP104440	H14	CIP104441
	KOG18	LP80	KOG18	LP80	KOG18	LP80
	ATCC8014	KOG24	KOG24	CIP104448	CIP1044448	ATCC8014
	LP85-2	NCIMB12120	DKO22	NCIMB12120	LP85-2	DKO22
	299v	299v	299	299	299v	299
	**NC8**	**NC8**	**NC8**	**NC8**	**NC8**	**NC8**

a Strains indicated in bold are consumed by all volunteers.

### DNA Isolation, Pyrosequencing, and Data Analysis of the Mixed Strains

DNA from *in vitro* bacterial cultures was extracted using InstaGene™ Matrix (Bio-Rad, Hercules, USA) according to the manufacturer’s instructions. For variable locus selection and intergenic region sequence determination, the DNA was amplified with primers A to V according to Table S2 and the resulting amplicons were purified using the Wizard® SV Gel and PCR Clean-Up System kit (Promega, Madison, USA), followed by sequencing (BaseClear, Leiden, The Netherlands). To visualize strain-specific variation in the intergenic region between *lp_0339* and *lp_0340*, the Clone Manager program (version 9.03, Scientific & Educational Software, Cary, USA) was used to align the sequences.

DNA isolation from feces was performed as previously described [34], [35]. Briefly, after bead-beating, DNA was purified by 2 to 3 phenol-chloroform extractions, followed by overnight precipitation of the DNA using 1 volume of isopropanol and 1/10 volume of sodium acetate. The resulting pellets were washed with 70% (v/v) ethanol, and dissolved in 100 µl TE buffer by overnight incubation at 4°C. All PCR reactions were performed using KOD Hot Start DNA polymerase (EMD Bioscience, Gibbstown, USA) according to the manufacturer’s instructions with primer combinations as listed in Table S2 and S3. The reverse primers used to generate amplicons for high-throughput sequencing of amplicons derived from DNA isolated from the fecal material harbored a unique 6 nt barcode, allowing discrimination of all the samples derived from different time-points and volunteers in a pooled amplicon mixture (Table S2). After amplification of the variable intergenic region from fecal DNA, the resulting amplicons were purified using the Invitek MSB® HTS PCRapace kit (STRATEC Molecular, Birkenfeld, Germany) and their concentrations were measured by NanoDrop (ND-1000 Spectrophotometer, NanoDrop Technologies, Wilmington, USA). Subsequently, the amplicons were pooled in equimolar amounts and ran on, and isolated from a 1.5% agarose gel using the Wizard® SV Gel and PCR Clean-Up System kit (Promega, Madison, USA), and analyzed by massive parallel sequencing on a GS FLX (titanium chemistry, GATC Biotech AG, Konstanz, Germany). Sequence data were binned per sampling time point on basis of the unique 6 nt barcodes using the Qiime pipeline [36]. Subsequently, for each of the sequences within a sample, the best hit was determined among the sequences of the 10 variable regions using BLAST [37] in combination with *ad hoc* Python scripts to quantify the relative amount of each strain, using the strictest sequence identity criteria possible (cutoff of 100% sequence identity across the barcode and the relevant region of the intergenic sequence). In total 89% of the sequences could be linked with a sample.

### Quantitative PCR to Determine *L. plantarum* Amounts

Quantitative PCR using SYBR Green was applied to determine total *L. plantarum* amounts or amounts of the 10 consumed *L. plantarum* strains with the *L. plantarum* 16S-specific primer pair Lp-16Sfo(2) plus Lp-16Sre(2) [28] (Table S2) or the intergenic locus-specific primers Q-PCR_10LP_strains_F plus Q-PCR_10LP_strains_R, respectively (Table S2). 1× Power SyberGreen (Molecular Probes, Eugene, USA), 10 pmol forward primer, 10 pmol reverse primer, and 1000- or 10,000-fold diluted DNA were used as starting material. Reactions were initiated at 95°C for 3 min, followed by 40 amplification cycles consisting of a denaturation step at 95°C for 15 sec, primer annealing at 50°C for 30 sec, and extension at 72°C for 30 sec. Similarly, for the determination of the 10 consumed *L. plantarum* strains, reactions were initiated at 50°C for 2 min and 95°C for 10 min, and followed by 40 amplification cycles consisting of a denaturation step at 95°C for 15 sec and primer annealing and extension at 60°C for 1 min. All runs were completed with amplicon-integrity verification by melting curve analysis. All reactions were performed using a 7500 Fast Real-Time PCR System (Applied Biosystems, Nieuwekerk a/d IJssel, The Netherlands). Cycle threshold values were obtained upon manual setting of the baseline at a threshold value at which fluorescence was appreciably above background and within the exponential phase of amplification for all reactions.

### Statistical Analyses and Strain Clustering

A Spearman's Ranktest was used to determine the correlation of the *L. plantarum* strains’ survival in the *in vitro* GI-tract assay using cells harvested from logarithmic phase compared to stationary phase-harvested cells. Furthermore, this test was used to determine the correlation of the *in vitro* GI-tract survival (stationary phase harvested) compared to the *in vivo* GI-tract persistence of the strains consumed by the first 5 subjects (Table 1). Strains were ranked for robustness according to their log_10_CFU/ml survival rate after 60 min of gastric juice incubation or according to the averaged difference in relative numbers of sequences after intake of all 5 subjects divided by the relative numbers of sequences of the input sample, respectively. The strains from the latter ranking only got a distinctive ranking if their average value of the different measurements was outside the standard deviation of the nearest strain, while if this was not the case, both strains received the same ranking. The statistical significance of differences between Spearman correlations was determined by Fisher's Z transformation, and *P*-values <0.05 were considered significant.

Hierarchical clustering of the individual *L. plantarum* strains based on their absence/presence of genes [19], [38] was performed using average linkage agglomeration and Pearson correlation in Genesis [39].

## Results

### A GI-tract Mimicking Assay Reveals Extensive Diversity in Survival of 42 *L. plantarum* Strains

**Figure 1:**
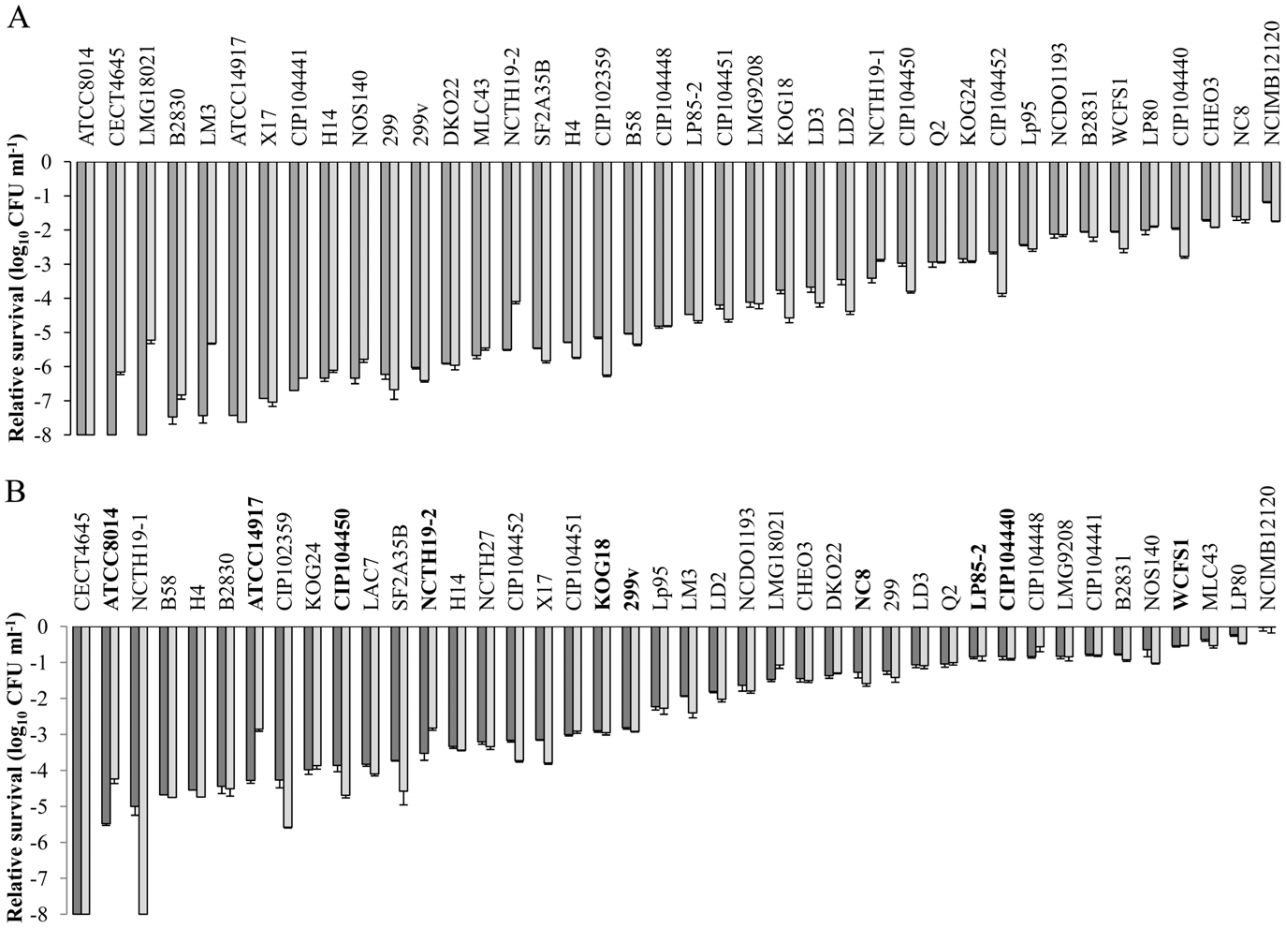
Relative survival of *L. plantarum* strains subjected to an *in vitro* GI-tract assay. **** Relative viability loss of *L. plantarum* strains harvested from logarithmic phase (panel A) or stationary phase (panel B) of growth after 60 min (dark grey) gastric juice incubation and subsequent 60 min (light grey) pancreatic juice incubation. The starting population size is set at 0 Log_10_ CFU ml^−1^, the data presented are averages of technical triplicates (+ standard deviation). Strains depicted in bold in panel B were present in the bacterial preparation consumed by subjects 1 to 5.

To determine the dynamic range of survival, 42 *L. plantarum* strains, including the reference strain WCFS1, were subjected to a GI-tract mimicking assay. This experiment revealed that the relative GI survival of the strains exceeded a 7 log_10_ CFU/ml difference for cells harvested either from the logarithmic or stationary phase of growth (Fig. 1). Cells harvested from the stationary phase commonly displayed higher survival compared to cells harvested from the logarithmic phase (Fig. 1A and B). Irrespective of the growth phase from which the cells where harvested, the best surviving strain was *L. plantarum* NCIMB12120, while strains ATCC8014 and CECT4645 displayed the lowest GI survival (Fig. 1). A positive and significant (p<0.01) correlation was observed between the strain-specific relative survival when sampled from the logarithmic phase or from the stationary phase, indicating that the differences in survival were independent of the growth phase. Notably, the reference strain WCFS1 was one of the better surviving strains as it was ranked as 6^th^ (logarithmic phase) and 4^th^ (stationary phase) most robust strain, displaying survival rates that were within 1-log_10_ difference relative to the most robust strain NCIMB12120 (Fig. 1).

To identify candidate genes of *L. plantarum* that affect GI-tract robustness, the survival data of each strain were correlated to genomic diversity data obtained by comparative genome hybridization (CGH) using *L. plantarum* WCFS1 as the reference genome [19]. The colony enumeration of the 42 *L. plantarum* strains (both for logarithmic and stationary phase cells) after exposure to the GI-tract assay conditions were correlated with the CGH derived diversity data using the random forest algorithm [40]. Unfortunately, these analyses did not reveal significant correlations between gene presence and absence patterns in individual strains in relation to their relative GI robustness. The genes that were identified by this correlation with the highest relative significance were consistently belonging to the *L. plantarum* prophages, which are known to be highly variable between strains [19], [38], and were considered not plausible as candidate effector-genes in relation to GI-tract survival.

### Discrimination of Mixed *L. plantarum* Strains on Basis of a Variable Intergenic Region

**Figure 2:**
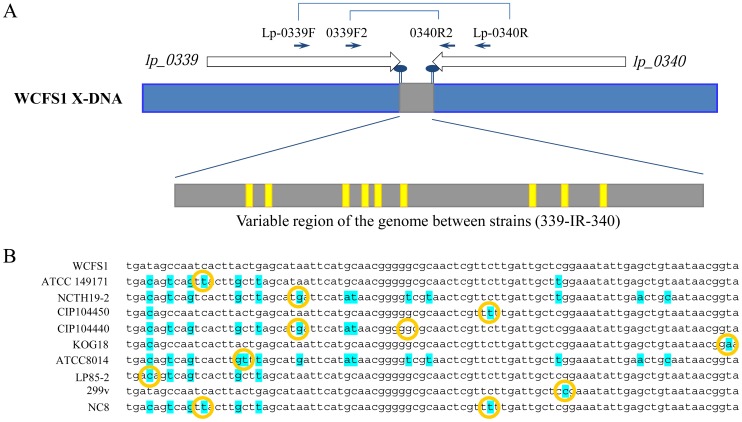
Schematic representation of the 339-IR-340 region of *L. plantarum* strains. Panel A: Schematic representation of the variable region (grey area) between the *lp_0339* and *lp_0340* genes (white open arrows) of *L. plantarum* WCFS1 with the single nucleotide polymorphism positions (yellow areas) detected in the other strains. Primers used to generate amplicons for sequencing are displayed. Panel B: Sequence comparison of the 10 sequence variations in the 339-IR-340 intergenic region. Yellow circles indicate the nucleotide(s) that distinguish the 339-IR-340 sequence types.

To enable assessment of the *in vivo* GI-tract persistence and survival of mixtures of *L. plantarum* strains, and to compare the obtained data to the *in vitro* results, we aimed to identify and exploit a variable region in the genomes of 40 *L. plantarum* strains. Notably, the 2 strains excluded in this analysis as compared to the *in vitro* assay presented above were isolated from spinal fluid or tooth abscess and were therefore considered unsuitable for the human volunteer study. As a source of anticipated variable DNA sequences, non-coding intergenic regions were explored based on the genome sequence of *L. plantarum* WCFS1 [22]. Candidate intergenic loci were selected on basis of (i) convergent orientation of the flanking genes, (ii) universal conservation of the flanking genes among the strains according to comparative-genome hybridization [38], (iii) length of intergenic region (150–200 bp) and (iv) absence of expression correlation of the flanking regions [41], [42]. Moreover, the candidate genetic loci were not allowed to be conserved in other species to prevent the targeting of conserved multi-gene loci. Eleven regions fulfilling these criteria were selected for design of degenerated primers based on the amino acids sequences of the proteins encoded by the flanking genes present in *L. plantarum* WCFS1 (Table S3). These degenerated primers (Table S2, Fig. 2A) were used for amplification of the intergenic regions by PCR using chromosomal DNA from at least 8 *L. plantarum* strains as a template. The target loci that yielded a single amplicon of a length comparable to that obtained with WCFS1 in at least 5 strains were subjected to amplicon sequencing. Some of the amplicons evaluated contained little variation between the strains and thereby were considered unsuitable for the purpose of sequence-based strain tracking, while other amplicons were excluded because their sequencing generated ambiguous results (Table S3). The intergenic region between *lp_0339* and *lp_0340* (designated 339-IR-340) satisfied all criteria mentioned above. To enhance amplification reliability, novel, non-degenerated primers were designed on basis of conserved nucleotide sequences within the amplicon sequences corresponding to the flanking genes of 339-IR-340 (Table S2, Fig. 2A). The isolated genomic DNA of the 40 *L. plantarum* strains was used as template in PCR reactions, resulting in 0.5 kb amplicons using template DNA derived from 34 strains. Subsequent sequencing of these amplicons revealed 10 distinct intergenic sequences in these 34 strains (Fig. 2B and Table S1).

**Figure 3:**
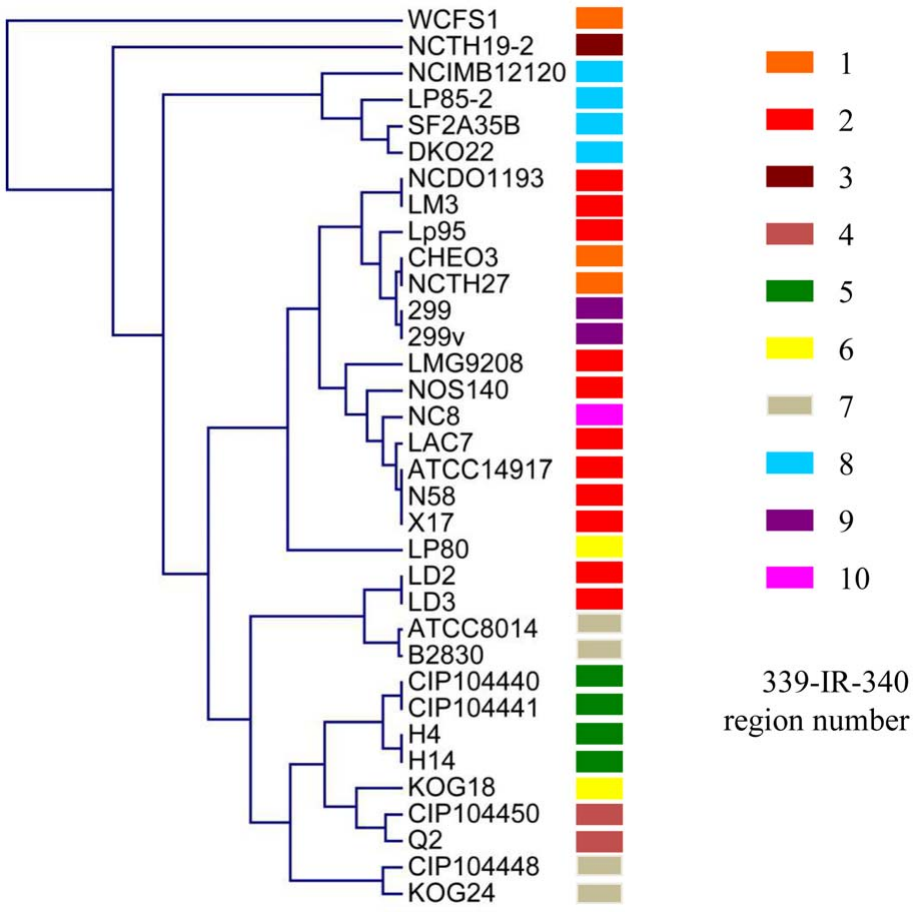
Co-clustering of 34 *L. plantarum* strains based on the presence/absence gene profiles and the 339-IR-340 region distribution. The previously published comparative genome hybridization datasets [19] were used to construct the genomic relatedness tree presented, which was complemented with the distribution of the 10 distinct 339-IR-340 sequence types, indicated by the colored bars.

To investigate the distribution of the different variable regions among these 34 strains, the 339-IR-340 regions were projected on the dendogram that was created on basis of the CGH data available for these strains [19], [38]. Only 4 of the different sequence variations of the 339-IR-340 region did not co-cluster with the subgroups of strains as they clustered together in the CGH-based dendogram (Fig. 3). This observation indicates that the strain-specific gene absence/presence distributions (based on CGH) are largely, but not universally, correlated with the sequence variation in the 339-IR-340 intergenic region selected. This variable sequence-tag present in the genomes of these strains of *L. plantarum* was employed for sequence based strain-specific quantification in strain-mixtures as described below.

Four mixtures were designed that each contained 10 *L. plantarum* strains with 10 distinctive 339-IR-340 sequences. Using the DNA isolated from these mixtures of 9 strains with a variable amount of the tenth strain (reference WCFS1), revealed that reproducibility of the relative contribution of the 9 strains to the overall bacterial preparation was very high (maximal 11% variation, Table 2). Moreover, the titration of different amounts of the reference strain WCFS1 in this mixture (10-fold dilution range) revealed that within a range of 100-fold dilution the relative abundance of this strain could still be assessed with high accuracy, while higher dilutions of the WCFS1 population appeared to lead to overestimation of the WCFS1 relative abundance as compared to its actual size (Fig. S1). These experiments establish that the amplicon sequence distribution data allow the accurate detection of strain-specific relative-abundance decreases within a community up to 100-fold, which was clearly sufficient for the reliable determination of strain-specific relative abundances in fecal samples (see below).

**Table 2:** Relative *L. plantarum* strain abundance of 4 independent replicates^a^.

Stain Nr^b^	ATCC14917	NCTH19-2	CIP104450	CIP104440	KOG18	ATCC8014	Lp85-2	299v	NC8	Total
1	0.143	0.188	0.059	0.079	0.120	0.194	0.029	0.099	0.088	1
2	0.140	0.206	0.046	0.083	0.118	0.200	0.028	0.096	0.084	1
3	0.142	0.183	0.050	0.091	0.125	0.195	0.028	0.097	0.088	1
4	0.144	0.187	0.054	0.080	0.121	0.182	0.034	0.100	0.098	1
Average	0.142	0.191	0.052	0.083	0.121	0.193	0.030	0.098	0.090	
St dev^c^	0.002	0.010	0.006	0.006	0.003	0.008	0.003	0.002	0.006	

a Four mixtures were designed that each contained 10 *L. plantarum* strains with 10 distinctive 339-IR-340 sequences. The variable amount of the tenth strain (reference WCFS1) was a dilution series and is subtracted from the other strains.

b Nr indicates sample number.

c St dev indicates standard deviation of the 4 replicates.

### Human Trial Setup

The size of the endogenous *L. plantarum* populations were determined in 2 fecal samples collected from each volunteer prior to initiation of the trial, using Q-PCR with total fecal-DNA as template with primers specific for the *L. plantarum* 16S rRNA gene [28]. The endogenous population of all subjects was on average 3.4 (±0.41) log_10_ ng/µg DNA. To assess the population dynamics of a single dosage of 10^11^ bacteria of a mixed population of *L. plantarum* strains in the GI-tract of healthy volunteers, mixtures were designed to contain 10 *L. plantarum* strains with 10 unique variable regions (Table 1). Subsequently, the abundance of individual *L. plantarum* strains was quantitatively monitored in fecal samples collected at different time-points after administration.

Five subjects received a preparation with an identical mixture of *L. plantarum* strains, to assess the variation in population dynamics in individual volunteers using a fixed input community. Next to this group of 5 subjects, the amount of strains that could be assessed in this human trial was enlarged by providing alternative mixtures of 10 *L. plantarum* strains that can be distinguished on basis of their 339-IR-340 sequence to the other 5 volunteers. Overall, this enabled the evaluation of competitive persistence of a total of 21 strains using a universal DNA amplification and sequence analysis regime. Notably, both the reference strain WCFS1 as well as the two strains (NCTH19-2 and NC8) that harbor unique 339-IR-340 sequences (Table S1) were included in all strain mixtures provided to the volunteers. These common strains functioned as reference strains to allow persistence evaluation of the 21 strains relative to these references (Table 1). Following administration, fecal sample collection was performed on a daily basis for a period of 10 days, as well as on days 14 and 21 after consumption. In addition, to determine whether all strains survived the digestive tract, DNA was isolated and amplified from plated fecal samples of 2 subjects (see materials and methods section for more details). These samples indicated that indeed all 10 strains survived GI passage (data not shown).

Q-PRC was used to determine the total *L. plantarum* community size, using primers designed on the universal part flanking the 339-IR-340 region of the 21 strains included in this study. The first fecal samples collected (usually obtained within 1.5 days after the bacterial mixture intake by the subjects) contained an approximately 2–3 log increased *L. plantarum* population. However, after 3 to 4 days, the *L. plantarum* population sizes returned to the levels prior to intake (data not shown). Fecal DNA samples from which amplicons could be generated were included in the amplicon pyrosequencing analysis. After barcode-based assignment of the sequence data to specific samples, the total numbers of sequences recovered per sample varied between 4805 to 16,905 sequence reads.

### Conserved GI-tract Persistence Patterns of *L. plantarum* Strains among Human Subjects

**Figure 4:**
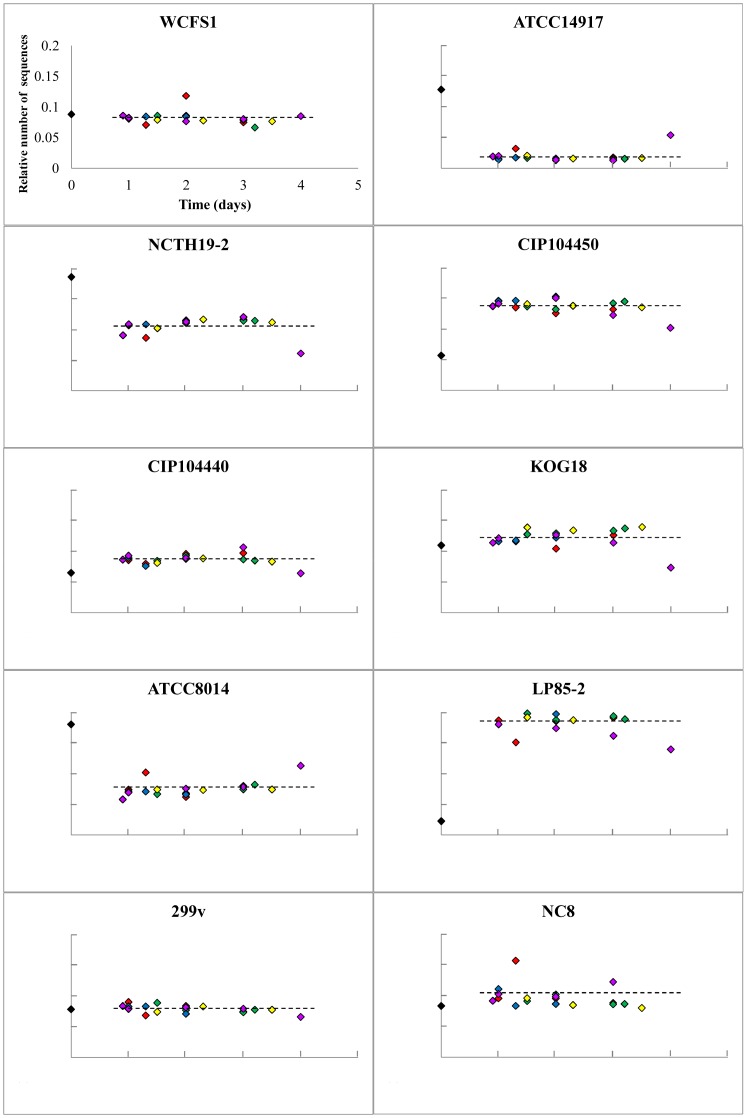
Strain-specific*L. plantarum* relative abundance after human consumption as detected by pyrosequencing. Relative strain abundances of the bacterial preparations consumed by the volunteers are depicted in black diamonds and those determined in time-specified post-consumption fecal material from the subjects 1 to 5 in red, green, blue, purple, and yellow diamonds, respectively. The graphs represent the number of strain specific sequences in the amplicons generated from DNA derived from fecal samples, divided by the number of strain-specific sequences identified in the input mixture amplicon. The total number of sequences per sample was set at 1 for normalization purposes. Axis-scaling in all the graphs is the same as depicted for strain WCFS1.

**Figure 5:**
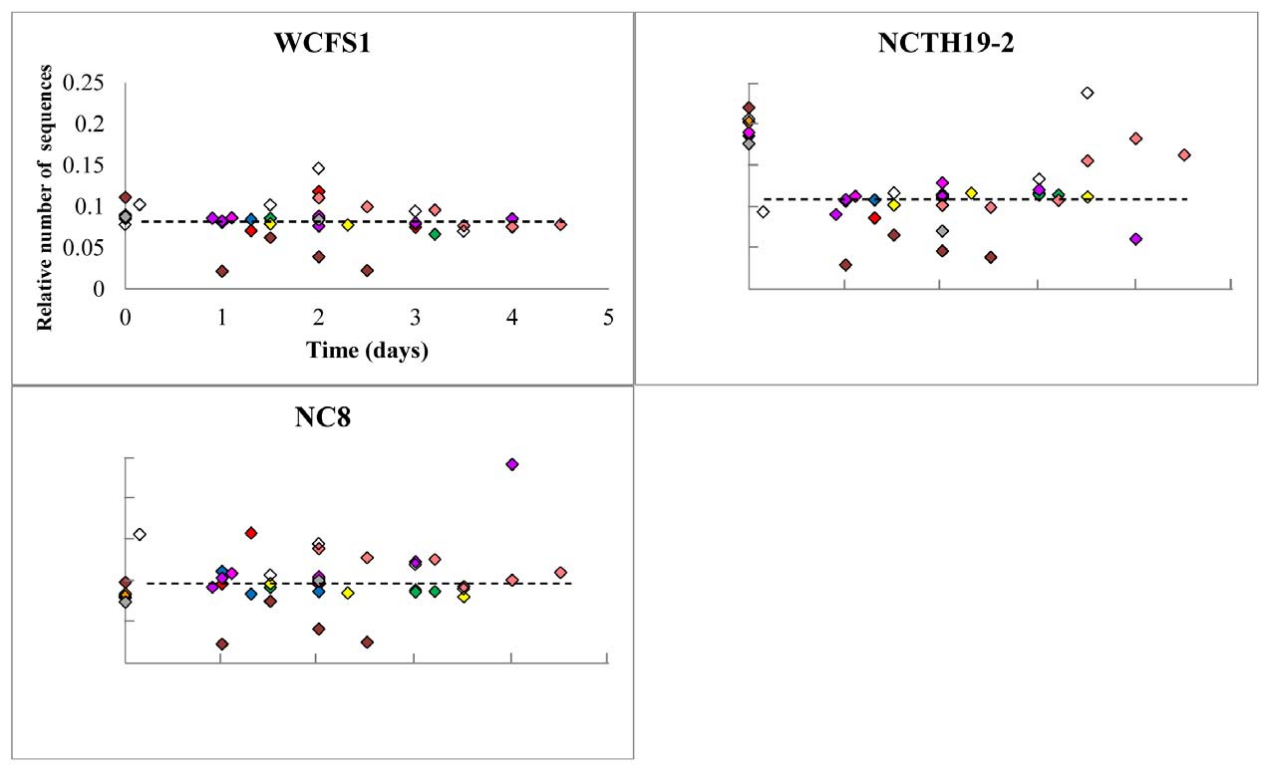
*L. plantarum* strain WCFS1, NCTH19-2, and NC8 relative abundance after human consumption as assessed by pyrosequencing. Relative strain abundances from subjects 1 to 10 are depicted in red, green, blue, purple, yellow, pink, brown, orange, white and grey diamonds, respectively. The graphs represent the number of strain specific sequences in the fecal amplicons, divided by the number of strain-specific sequences identified in the input mixture amplicon. The total number of sequences per sample was set at 1 for normalization purposes. Axis-scaling in all the graphs is the same as depicted for strain WCFS1.

Initially focusing on the 5 volunteers who consumed the same mixture of strains, it appeared that in all volunteers a consistent group of 5 strains in this mixture were recovered in an approximately equal relative abundance as compared to the input mixture (Fig.4). In contrast, the strains CIP104450 and Lp85-2 were recovered in substantially higher relative amounts as compared to their relative abundance in the input mixture. Conversely, strains ATCC14917, NCTH19-2, and ATCC8014 appeared to be underrepresented in the fecal output compared to their abundance in the input mixture (Fig. 4). Remarkably, the *L. plantarum* community composition remained virtually identical over time in all 5 subjects (Fig. 4). Moreover, evaluation of the relative abundance of the 3 strains that were consumed by all 10 volunteers revealed that, although the variation was larger compared to the 5 subjects who consumed the fixed strain mixture, the same trend was observed for these strains, i.e., WCFS1 and NC8 were stable over time, whereas the relative abundance of NCTH19-2 decreased consistently compared to the input mixture (Fig. 5).

Evaluation of the strain-specific abundance profiles obtained from the other 5 subjects (6–10) that consumed variable *L. plantarum* mixtures, revealed that, despite the small sample numbers, consistent observations were made with respect to the relative abundance of particular strains in the fecal preparations in comparison to their abundance in the corresponding input mixture (Fig. S2A–E). For example, strains LD3, NCIMB12120, and DKO22 seemed to be consistently present in increased amounts compared to their relative population size in the input mixture. In contrast, KOG24, CIP10448, and Lp80 were consistently recovered in smaller relative amounts in comparison to their relative abundance in the input mixture (Fig. S2A–E). Strain Lp95 was administered in mixtures provided to subject 10 and 6, and was recovered in relative high amounts in fecal populations analyzed for subject 10, but was only recovered with relatively low abundance from fecal material of subject 6 (Fig. S2A and E). Notably, strain DKO22 that belongs to the ssp. *argentoratensis* [43] and was consumed by subjects 7 and 10 was detected as the strain with the highest relative abundance increase among all strains tested in this study (Fig. S2B and E), which exceeded the increasing relative abundance described for strains CIP104450 and Lp85-2 (see above).

### Correlation of *in vivo* and *in vitro* GI-tract Persistence Profiles

As the magnitude of the effect on strain specific survival/persistence is considerably different between the *in vitro* and *in vivo* analyses, the ranking of the persistence of individual strains was compared using a Spearman’s rank test. This statistical analysis revealed that the *in vivo* strain persistence of the strains from the fixed strain mixture and their *in vitro* GI-tract survival (harvested from stationary phase, Fig. 1B) were positively and significantly (*p* = 0.001) correlated, demonstrating the predictive value of the *in vitro* assay for the pre-selection of strains that are anticipated to display relatively high persistence in the human GI tract. Overall, these data indicate that there are conserved persistence patterns in human individuals that are strain specific, and that the relative persistence may be qualitatively predicted using the simplified *in vitro* screening model presented here.

## Discussion

Our *in vitro* GI-tract assay revealed that individual *L. plantarum* strains displayed dramatic differences in GI-tract survival. These data expand earlier *in vitro* observations of variation of GI-robustness among small numbers of *L. plantarum* strains [14], [15], towards an extensive cohort of strains of this species that were isolated from various geographical locations and diverse habitats [19]. Considerable variations between *L. plantarum* strains have been reported for other phenotypes as well, such as degradation of carbohydrates, growth at 45°C, and tolerance to NaCl or nisin in the growth medium [19]. Despite the reported success of CGH approaches for the identification of the genetic basis for phenotypes such as mannose specific adhesion and the immunomodulatory capacities of *L. plantarum* [44]–[46], no significant and plausible correlations between gene presence and absence patterns in individual strains was revealed in relation to their relative GI robustness. This finding suggests that the differences in GI-tract survival are unlikely to be caused by the absence or presence of specific genes compared to the reference strain *L. plantarum* WCFS1. Consequently, it seems likely that the survival differences in the GI-tract assay are predominantly determined by differential gene expression levels of genes that are conserved among the strains included in this collection [19]. This notion is also supported by a recent study performed in our laboratory that demonstrated that the *L. plantarum* WCFS1 GI-tract robustness can be correlated to the transcription level of specific genes [7].

To determine competitive *in vivo L. plantarum* persistence, the variable intergenic region 339-IR-340 was used to develop a novel, high-throughput method to study the population dynamics of mixtures of strains in (complex) matrices like feces. Methods that were already available to discriminate *in vivo* digestive tract survival of specific strains in a mixture include selective plating of fecal samples followed by confirmation of strain/species identity, e.g. by methods based on physiological characteristics like sugar utilization capacity [15]. Alternative discriminatory methods rely on molecular typing techniques like plasmid or genomic DNA profiling using restriction enzyme analysis (REA) [15], pulsed-field gel electrophoresis (PFGE) [47], [48], or PCR based fingerprinting techniques like random amplification of polymorphic DNA (RAPD) [49], arbitrarily primed PCR (AP-PCR) [14], PCR-denaturing gradient gel electrophoresis (PCR-DGGE) [14], internal transcribed spacer PCR (ITS-PCR) [47], or Real-Time PCR [14], [50]. Generally, these techniques are labor-intensive and cannot be applied in a high-throughput manner. Alternative methods that can quantitatively discriminate individual strains in a large set of closely related mixed strains (e.g. from the same species) depend on introduction of different antibiotic resistance markers in the genome [51] or on discriminative insertions in the DNA (for example tags [24] or transposons [52]) in closely related strains. The method described here is analogous to the traditional multi-locus sequence typing (MLST), which relies on the natural genetic variance between strains. However, the method employed here targets an intergenic region with a high degree of sequence variability among strains rather than the commonly applied targets of housekeeping protein encoding genes in MLST. The intergenic region used here displayed 10-different sequence types among the strains analyzed but its sequence diversity may be expanded by sequencing this region in a larger panel of strains. Importantly, the method described here is compatible with barcoded next-generation sequencing for the quantitative determination of strain specific abundance levels in a complex mixture enabling low labor intensity, high-throughput analysis of community dynamics.

The detection of the 10 strains in the feces after consumption by healthy human volunteers via plating and pyrosequencing showed that all these strains are able to survive GI passage. Several studies have used inert radiopaque markers to establish that the upper limit of total GI transit time in normal individuals is 96 hour [53], [54]. The GI persistence of *L. plantarum* WCFS1 in human volunteers appeared similar to what has been detected before, i.e., detectable up to 3, but not up to 7 days after the last intake [13]. The shape of the persistence curve obtained for all *L. plantarum* strains also reflects the passage of *Bacillus stearothermophilus* spores that are considered to pass the intestine inertly [13]. Despite the typical transient behavior of *L. plantarum* in the human intestine, it is still very possible that *L. plantarum* influences the host, for instance by stimulating the immune system as has been demonstrated for different lactobacilli *in vivo*, including *Lactobacillus plantarum* [33], [55].

Remarkably, the persistence of individual strains appeared to be strongly conserved between human individuals. This suggests that intestinal passage is not drastically influenced by the subject-specific characteristics, such as gender, dietary intake, or endogenous microbiota composition. Moreover, the equal distribution of the 3 strains that were consumed by all volunteers indicates that the persistence is independent of the combination of *L. plantarum* strains used in the bacterial preparations. Although only measured in two volunteers, the strain with the most distinguishable enhanced persistence compared to the rest of the strains was DKO22. Intriguingly, the strains that cluster together on basis of their gene content with DKO22, namely NCIMB12120 and Lp85-2, also displayed a higher persistence as compared to the majority of the strains. These 3 strains all belong to the ssp. *argentoratensis* [43], suggesting that this subspecies may display enhanced GI persistence relative to the *L. plantarum* strains. A larger group of spp. *argentoratensis* strains should be tested to get a more accurate impression of the strain-specific GI-tract persistence of representatives of this subspecies.

The most discriminative factor involved in the determination of gut-persistence of *L. plantarum* consistently appears to be their capacity to survive the acid conditions encountered in the stomach. Following the loss of viability of the individual strains in the stomach mimicking conditions of the *in vitro* GI-tract assay, the subsequent small intestine-like conditions did not appear to drastically influence viability. This characteristic is also reflected by the recovery curve obtained in the *in vivo* persistence analysis in humans, where the strains all displayed identical recovery/persistence curves, suggesting that once they have passed the stomach, the rest of the intestinal tract does not provide any strain-discriminative selection conditions. Apparently the combination of strains in the mixture did not influence the survival capacity of its individual components, which is remarkable since competition is commonly expected to especially affect closely related strains. This observation may be related to the fact that *L. plantarum* is apparently not an effective colonizer of the intestinal tract of humans, and displays persistence curves that resemble that of a mere passant of the GI-tract, for which the gastric pH is the main hurdle for survival of intestinal passage.

The work presented here demonstrates that there is considerable variation in strain-specific GI-tract survival among *L. plantarum* strains, which is especially apparent from the *in vitro* assay results. These differences were substantially smaller in the *in vivo* persistence analysis, but the two approaches generated a congruent relative ranking of strains with respect to their GI-tract survival and/or persistence. Remarkably, the data presented imply that the *in vivo* persistence of *L. plantarum* strains is not strongly affected by the undoubtedly substantially different host-specific factors, like gender, genetic background, life-style and/or dietary habits.

## Supporting Information

Figure S1 **Input mixture administered to subjects 1–5, combined with 10-fold dilution range of** ***L. plantarum*** **WCFS1 relative abundance.** The reference strain WCFS1 was mixed in these standard mixtures in a 10-fold dilution range, and its strain specific detection is presented for a total of 4 10-fold dilution steps, starting with the relative abundance present in the mixture provided to subjects 1–5 in the human study. The relative number of sequences is depicted for WCFS1 (grey bars) and the other 9 strains (white bars, see Table 1). Total number of sequences per sample is set at 1.
(TIF)

Figure S2 **Strain-specific** ***L. plantarum*** **relative abundance after human consumption as detected by pyrosequencing.** Relative strain abundances of the bacterial preparations consumed by volunteer 6, 7, 8, 9 and 10 are individually presented in Fig. S2A, S2B, S2C, S2D and S2E, respectively.
(PPTX)

Table S1 **Strains used in this study.**
(DOC)

Table S2 **Primers used in this study.**
(DOC)

Table S3 **Primer pair combinations used for intergenic variable region amplification and summary of the subsequent PCR and sequencing results.**
(DOC)
