# Supplementary figures and images for: Congruent Strain Specific Intestinal Persistence of Lactobacillus plantarum in an Intestine-Mimicking In Vitro System and in Human Volunteers
Source: PLoS One. 2012 Sep 6;7(9):e44588. doi: 10.1371/journal.pone.0044588 (PMC3435264; doi:10.1371/journal.pone.0044588)

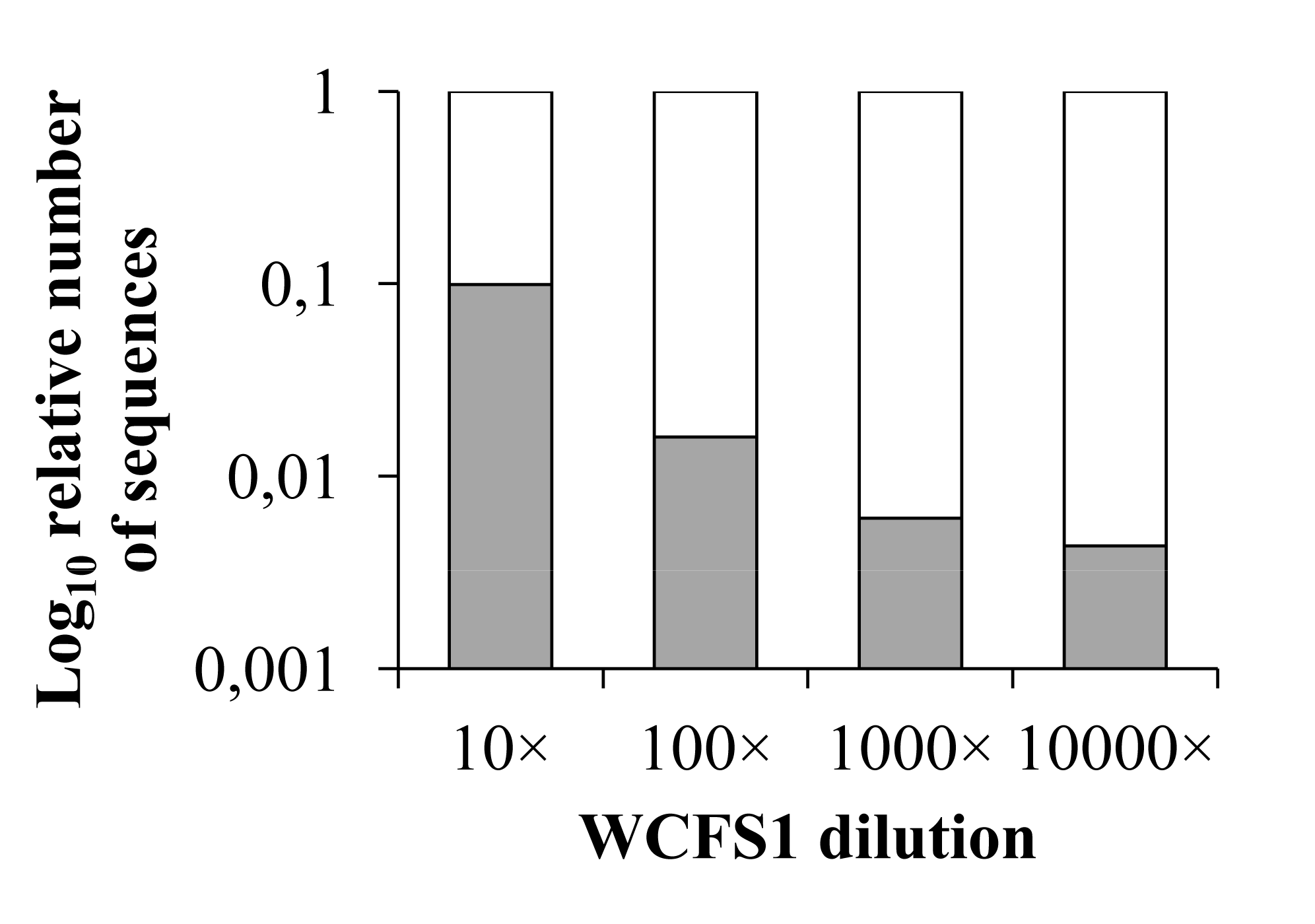

Supplement: Figure S1 — Input mixture administered to subjects 1–5, combined with 10-fold dilution range of L. plantarum WCFS1 relative abundance. The reference strain WCFS1 was mixed in these standard mixtures in a 10-fold dilution range, and its strain specific detection is presented for a total of 4 10-fold dilution steps, starting with the relative abundance present in the mixture provided to subjects 1–5 in the human study. The relative number of sequences is depicted for WCFS1 (grey bars) and the other 9 strains (white bars, see Table 1). Total number of sequences per sample is set at 1. (TIF) [file pone.0044588.s001.tif]

## Slide 1
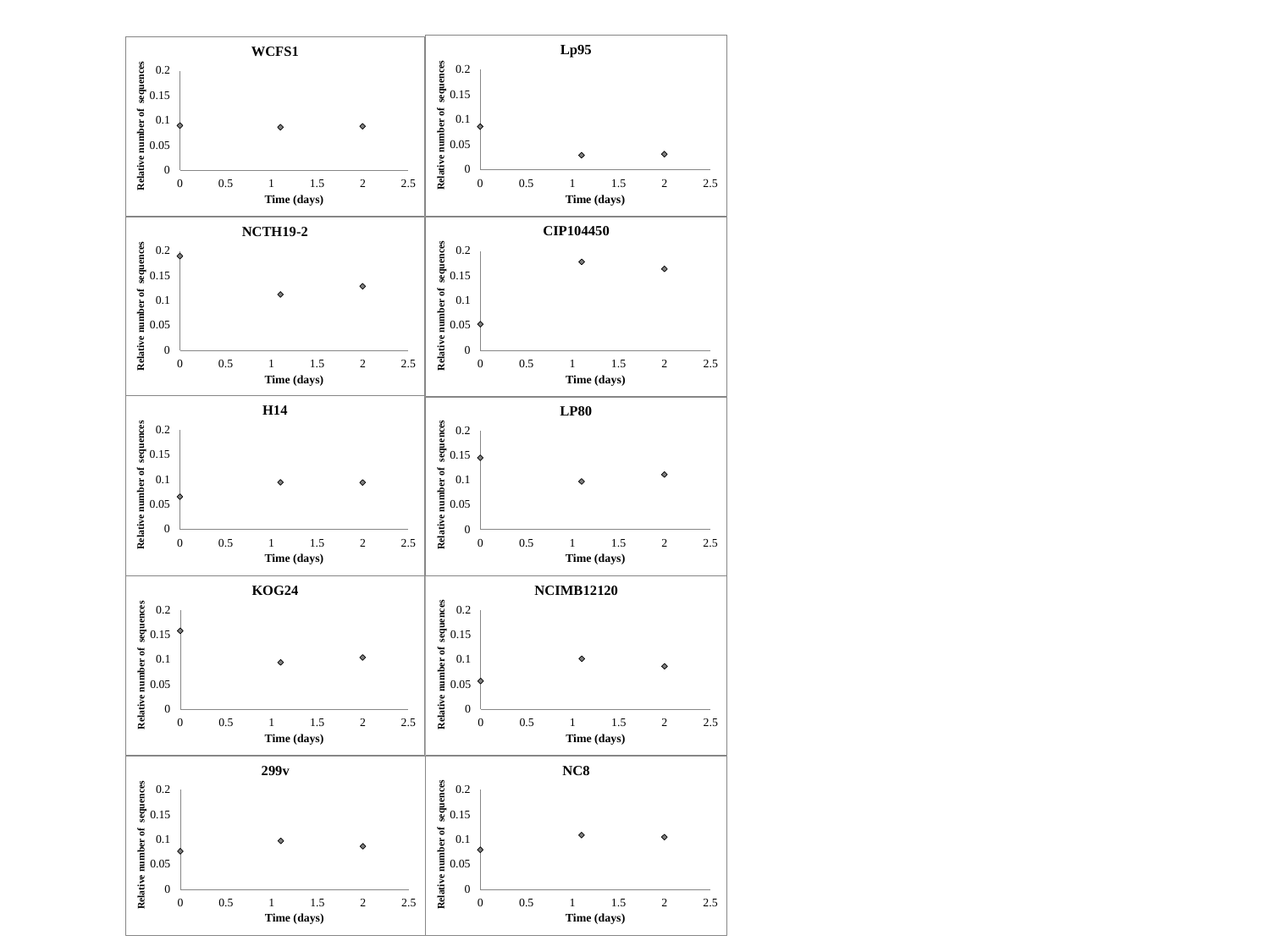

## Slide 2
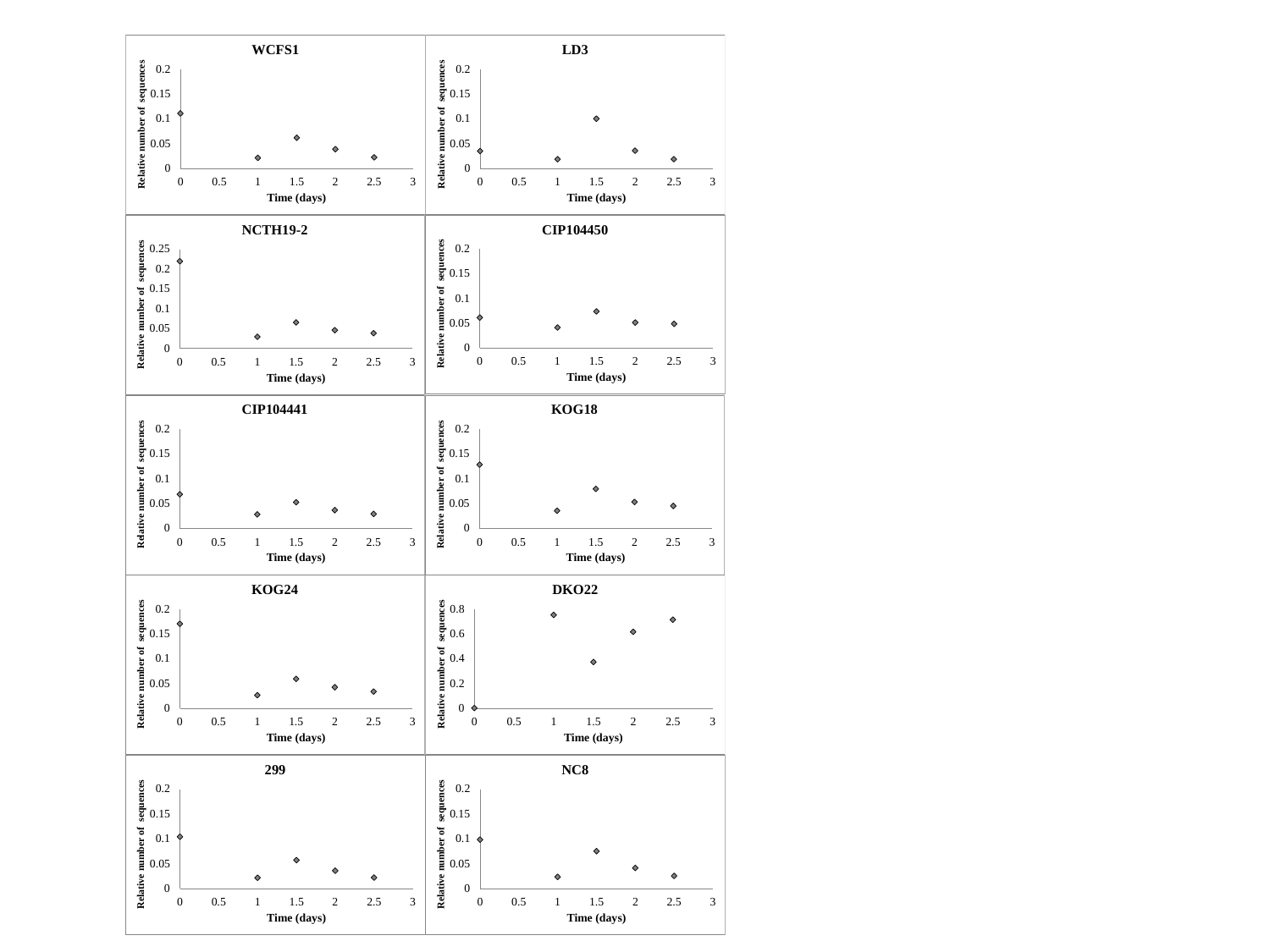

## Slide 3
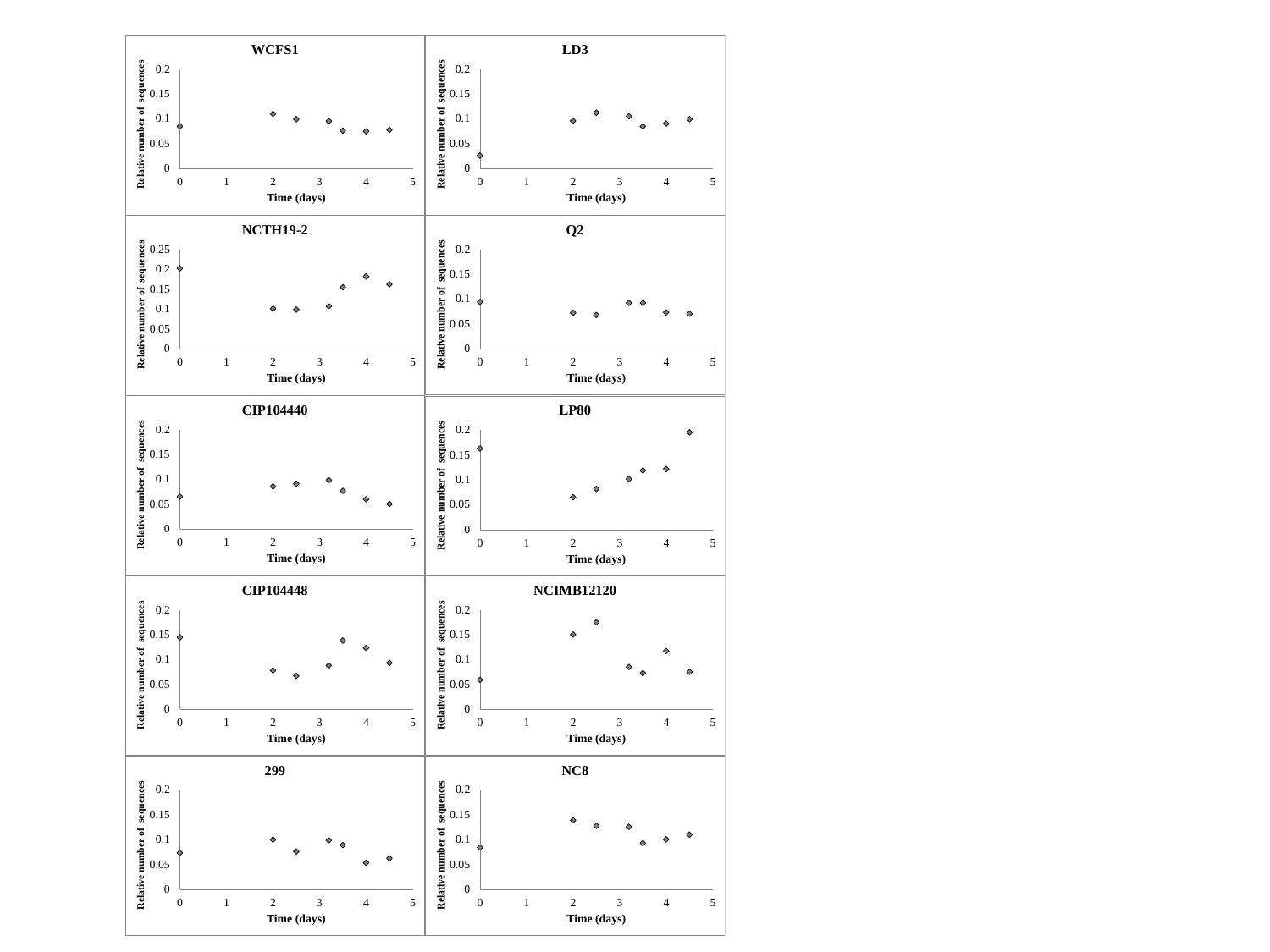

## Slide 4
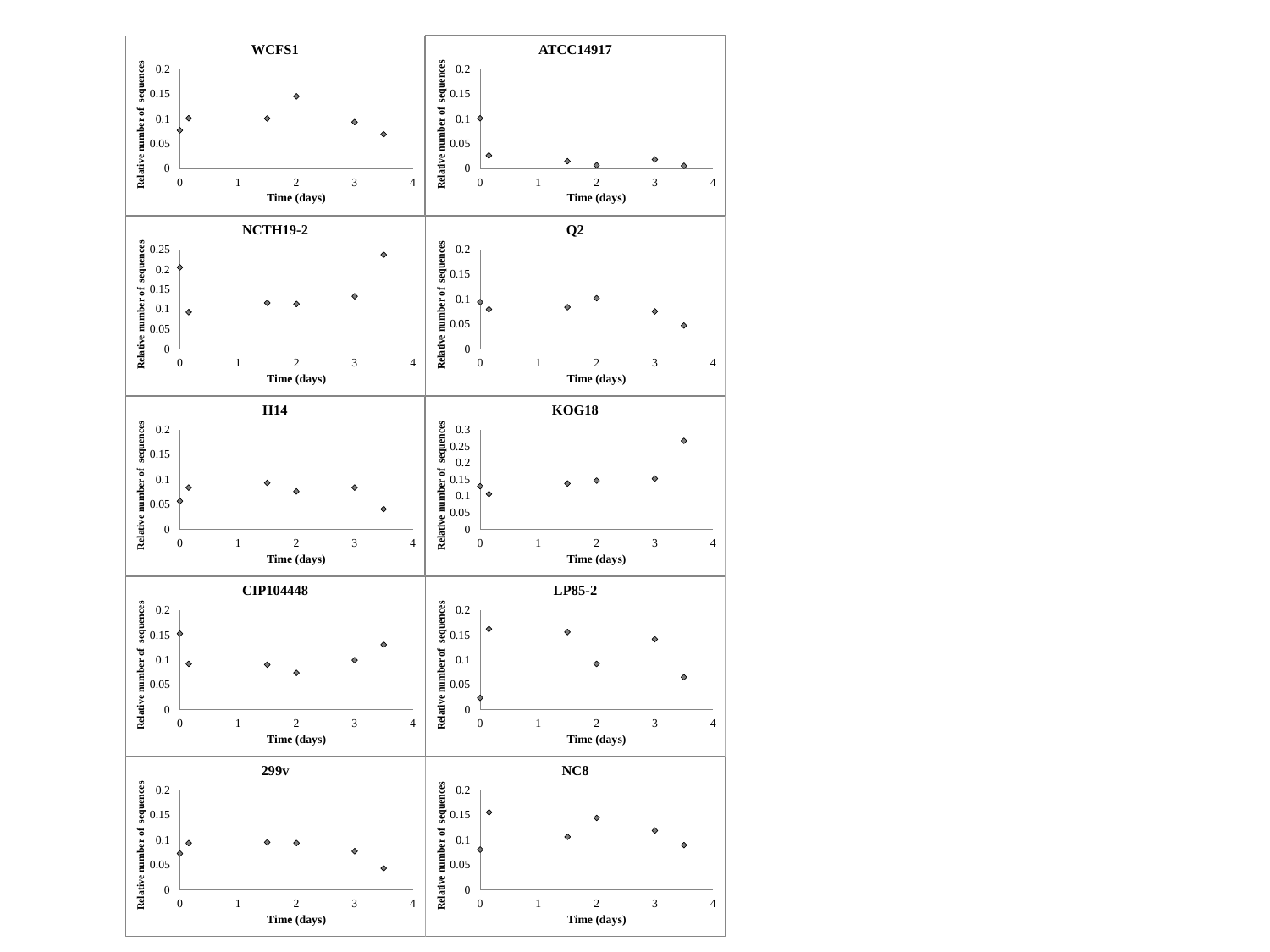

## Slide 5
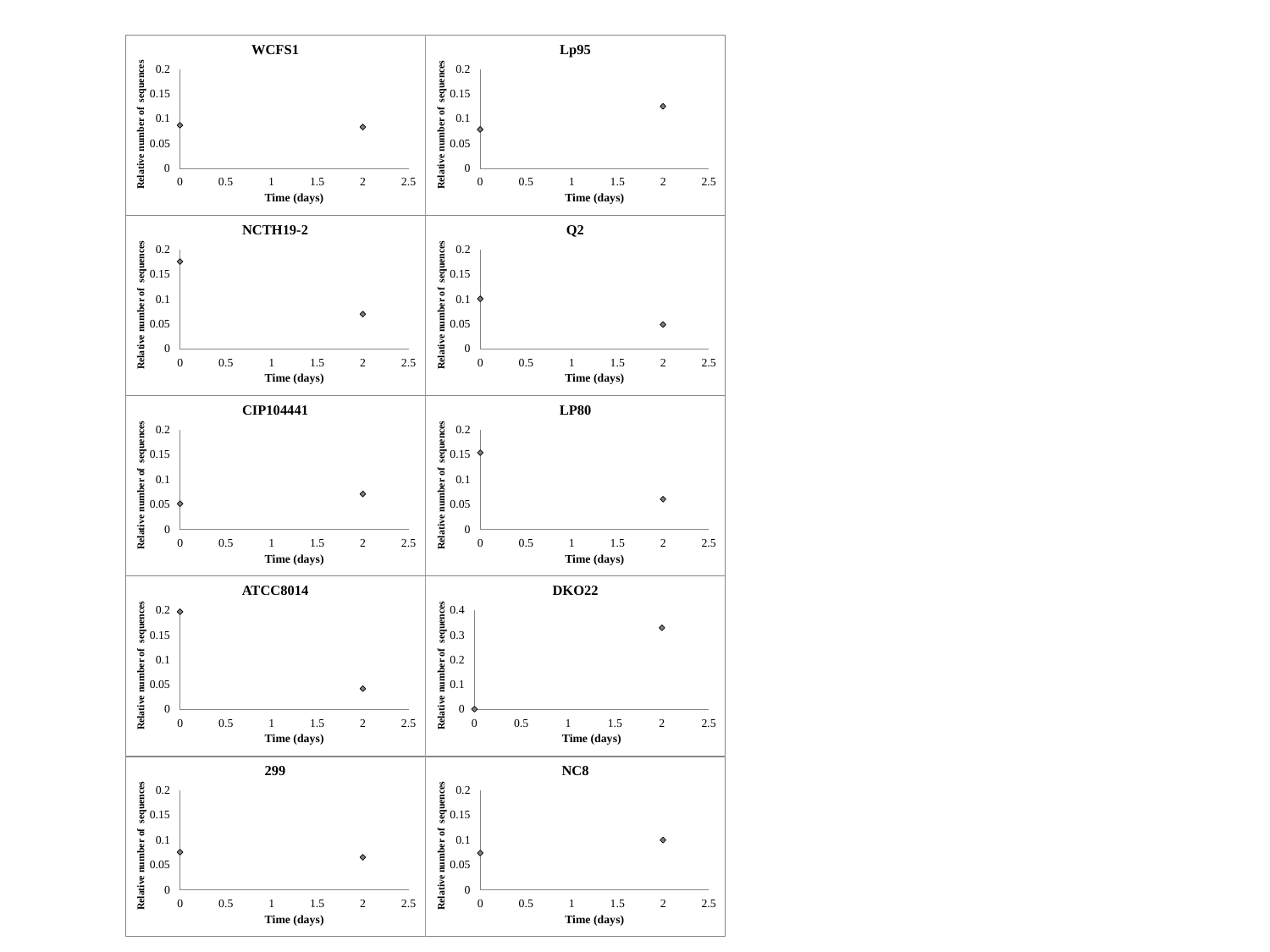

Supplement: Figure S2 — Strain-specific L. plantarum relative abundance after human consumption as detected by pyrosequencing. Relative strain abundances of the bacterial preparations consumed by volunteer 6, 7, 8, 9 and 10 are individually presented in Fig. S2A, S2B, S2C, S2D and S2E, respectively. (PPTX) [file pone.0044588.s002.pptx]
