# Supplementary material for: Congruent Strain Specific Intestinal Persistence of Lactobacillus plantarum in an Intestine-Mimicking In Vitro System and in Human Volunteers
Source: PLoS One. 2012 Sep 6;7(9):e44588. doi: 10.1371/journal.pone.0044588 (PMC3435264; doi:10.1371/journal.pone.0044588)
Supplement: Table S1 — Strains used in this study. (DOC) [file pone.0044588.s003.doc]

Table S1. **Strains used in this study^a^.**

E

D

C

B

| **Strain** | **Alternative designation** | **Origin** | **339-IR-340 region #**^b^ | **Source or reference**^c^ |
| --- | --- | --- | --- | --- |
| **WCFS1**^d^ | NCIMB 8826 | Human saliva, UK | 1 |  |
| **ATCC14917** | LMG 6907 | Pickled cabbage, Denmark | 2 | ATCC |
| MLC43 |  | Raw cheese with rennet, Italy |  | WUR |
| CHEO3 |  | Pickled sour sausage, Vietnam | 1 | NIZO |
| NCTH19-1 |  | Pickled sour sausage, Vietnam |  | NIZO |
| **NCTH19-2** |  | Pickled sour sausage, Vietnam | 3 | NIZO |
| NCTH27 |  | Pickled sour sausage, Vietnam | 1 | NIZO |
| LD2 |  | Fermented orange, Vietnam | 2 | NIZO |
| NOS140 |  | Cabbage kimchi, Japan | 2 | NIZO |
| **Q2** |  | Fermented sourdough, Italy | 4 | DSDA |
| H4 |  | Fermented sourdough, Italy | 5 | DSDA |
| **H14** |  | Fermented sourdough, Italy | 5 | DSDA |
| CECT4645 |  | Cheese |  | NIZO |
| **KOG18** |  | Turnip pickled with rice bran, Japan | 6 | NIZO |
| **KOG24** |  | Cheese, Japan | 7 | NIZO |
| LMG9208 |  | Sauerkraut, UK | 2 | NIZO |
| **Lp95** |  | Wine red grapes, Italy | 2 | NIZO |
| B2830 |  | Cassava sour | 7 | NIZO |
| B2831 |  | Cassava sour |  | NIZO |
| N58 |  | Pickled sour sausage, Vietnam | 2 | FIRI |
| X17 |  | Hotdogs, Vietnam | 2 | NIZO |
| LAC7 |  | Banana fermented, Vietnam | 2 | NIZO |
| **LD3** |  | Radish pickled, Vietnam | 2 | NIZO |
| **DKO22**^e^ |  | Cassava sour, Nigeria | 8 | NIZO |
| **299** | DSM 6595 | Human colon, UK | 9 |  |
| **CIP104440** | 61A | Human stool, France | 5 | CIP |
| SF2A35B^d^ |  | Sour cassava, South America | 8 |  |
| **NCIMB12120**^e^ |  | Ogi, Nigeria | 8 | NCIMB |
| **CIP104441** | 61P | Human stool, France | 5 | CIP |
| **CIP104450** | 61BR | Human stool, France | 4 | CIP |
| CIP104451 | 61CA | Human urine, France |  | CIP |
| CIP104452 |  | Human tooth abcess, France |  | CIP |
| **299v** | DSM 9843 | Human intestine, UK | 9 |  |
| **NC8** |  | Grass silage, Sweden | 10 |  |
| LM3 |  | Silage | 2 |  |
| **LP80** | DSM 4229 | Silage | 6 | C. Platteeuw |
| **LP85-2**^e^ |  | Silage, France | 8 |  |
| **ATCC8014** | LMG 1284 | Maize ensilage | 7 | ATCC |
| NCDO1193 | LMG 9209 | Vegetables | 2 | NCIMB |
| CIP102359 |  | Human spinal fluid, France |  | CIP |
| **CIP104448** | 61BB | Human stool, France | 7 | CIP |
| LMG18021 |  | Milk, Senegal |  | BCCM |

^a^ Adapted from Molenaar *et al*. and Siezen *et al*. .

^b^ Number of the variable intergenic region between *lp_0339* and *lp_0340*. The number is the same as in Figure 3.

^c^ NCIMB, National Collections of Industrial, Marine and Food Bacteria, United Kingdom; ATCC, American Type Culture Collection, USA; WUR, Wageningen University and Research Center, the Netherlands; NIZO, NIZO food research collection, the Netherlands; DSDA, Dipartimento di Scienza degli Alimenti, Universitá degli Studi di Napoli Federico, Italy; FIRI, Food Industries Research Institute, Vietnam; CIP, Collection of Institute Pasteur, France; and BCCM, Belgian Co-ordinated Collections of Micro-organisms, Belgium.

^d^ Strains in bold are consumed by the subjects, as they could be discriminated on basis of their 339-IR-340 region.

^e^ Putative subspecies *argentoratensis* .

**References**

1. Kleerebezem M, Boekhorst J, van Kranenburg R, Molenaar D, Kuipers OP, et al. (2003) Complete genome sequence of *Lactobacillus plantarum* WCFS1. Proc Natl Acad Sci U S A 100: 1990-1995.

2. Molin G, Jeppsson B, Johansson ML, Ahrne S, Nobaek S, et al. (1993) Numerical taxonomy of *Lactobacillus* spp. associated with healthy and diseased mucosa of the human intestines. J Appl Bacteriol 74: 314-323.

3. Figueroa C, Davila AM, Pourquié J (1995) Lactic acid bacteria of the sour cassava starch fermentation. Letters in Applied Microbiology 21: 126-130.

4. Johansson ML, Molin G, Jeppsson B, Nobaek S, Ahrne S, et al. (1993) Administration of different *Lactobacillus* strains in fermented oatmeal soup: *in vivo* colonization of human intestinal mucosa and effect on the indigenous flora. Appl Environ Microbiol 59: 15-20.

5. Aukrust T, Blom H (1992) Transformation of *Lactobacillus* strains used in meat and vegetable fermentations. Food Research International 25: 253-261.

6. Muscariello L, Marasco R, De Felice M, Sacco M (2001) The functional *ccpA* gene is required for carbon catabolite repression in *Lactobacillus plantarum*. Applied and Environmental Microbiology 67: 2903-2907.

7. Bringel F, Curk MC, Hubert JC (1996) Characterization of lactobacilli by Southern-type hybridization with a *Lactobacillus plantarum pyrDFE* probe. Int J Syst Bacteriol 46: 588-594.

8. Molenaar D, Bringel F, Schuren FH, de Vos WM, Siezen RJ, et al. (2005) Exploring *Lactobacillus plantarum* genome diversity by using microarrays. J Bacteriol 187: 6119-6127.

9. Siezen RJ, Tzeneva VA, Castioni A, Wels M, Phan HT, et al. (2010) Phenotypic and genomic diversity of *Lactobacillus plantarum* strains isolated from various environmental niches. Environ Microbiol 12: 758-773.

10. Bringel F, Castioni A, Olukoya DK, Felis GE, Torriani S, et al. (2005) *Lactobacillus plantarum* subsp. *argentoratensis* subsp. nov., isolated from vegetable matrices. International Journal of Systematic and Evolutionary Microbiology 55: 1629-1634.
