# Supplementary material for: Congruent Strain Specific Intestinal Persistence of Lactobacillus plantarum in an Intestine-Mimicking In Vitro System and in Human Volunteers
Source: PLoS One. 2012 Sep 6;7(9):e44588. doi: 10.1371/journal.pone.0044588 (PMC3435264; doi:10.1371/journal.pone.0044588)
Supplement: Table S2 — Primers used in this study. (DOC) [file pone.0044588.s004.doc]

Table S2. **Primers used in this study.**

| **ID** | **Name^a^** | **Sequence (5’ to 3’)^b^** | **Subject or sample description** | **Time after intake (days)^c^** |
| --- | --- | --- | --- | --- |
| A | Lp-0166R | CCCCARTGDGCNGGWTCRTGWCC | 166-IR-168 |  |
| B | Lp-0168R | DGCRTGDGHNGGYTCRTGWCC | 166-IR-168 |  |
| C | Lp-0339F | CNTWYAAYATGGCDGGNTGGCG | 339-IR-340 |  |
| D | Lp-0340R | GCCNGCNATGACNGGNTAYCCNGG | 339-IR-340 |  |
| E | Lp-0396F | ATNCCYTGRTGCCARTGNGGNGC | 396-IR-397 |  |
| F | Lp-0397R | HNCVCCAGCNADNGGNCGNCC | 396-IR-397 |  |
| G | Lp-0415F | GTATTCTTTGCAGATGGGGGC | 415-IR-416 |  |
| H | Lp-0416R | TAGTGTCATCCAAGATAGCTCC | 415-IR-416 |  |
| I | Lp-0587F | GGTGTTTGCGCAGAAAGTCCC | 587-IR-588 |  |
| J | Lp-0588R | YTGAATCCAYTCRTCRYTRGTRTCC | 587-IR-588 |  |
| K | Lp-0631F | TTCTTCNGTAAGATCTTCACCYCC | 631-IR-632 |  |
| L | Lp-0632R | CCAACACTWGGTGTTCTATGHCC | 631-IR-632 |  |
| M | Lp-2464R | TCRCTMGCDATAATGTTAATYGCHGC | 2464-IR-2466 |  |
| N | Lp-2466F | GTDAAAGCDATCGCTTWTGACCC | 2464-IR-2466 |  |
| O | Lp-2602R | ACRTAHTKTTGHTGATTDAWVACRCG | 2602-IR-2603 |  |
| P | Lp-2603R | AAATCACGAAACCCATGAAACCC | 2602-IR-2603 |  |
| Q | Lp-3124R | CAATATCCTGAGCAGTGCCC | 3124-IR-3125 |  |
| R | Lp-3125R | CGGCTTCTAGGGCTGCCGC | 3124-IR-3125 |  |
| S | Lp-3233R | AAATCAAACGAAATGAGCGCCC | 3233-IR3234 |  |
| T | Lp-3234R | CTACGGTAATGGGCGAGAGC | 3233-IR3234 |  |
| U | HlociF1 | TTAGTTGTTCAGATTCCAGGC | Hloci-IR-Hloci |  |
| V | HlociR1 | CCCTGGTACAATGGGACC | Hloci-IR-Hloci |  |
| W | 0339F2 | CGCCGTAATCAGTTCTTTACG | 339-IR-340 |  |
| X | 0340R2 | CCTTTGGGTACATGGACGCG | 339-IR-340 |  |
| PS00 | PS.001 B lp_0339f HvB^d^ | CCTATCCCCTGTGTGCCTTGGCAGTCTCAGTATACCAGTGAAGCATTTGCCG | All subjects | All |
| PS01 | PS.001 A lp_0340r HvB | CCATCTCATCCCTGCGTGTCTCCGACTCAGccaataGCGTACCTGTTAGAGAAGCGG | 1 | 1 |
| PS02 | PS.002 A lp_0340r HvB | CCATCTCATCCCTGCGTGTCTCCGACTCAGccacaaGCGTACCTGTTAGAGAAGCGG | 1 | 2 |
| PS03 | PS.003 A lp_0340r HvB | CCATCTCATCCCTGCGTGTCTCCGACTCAGccacgcGCGTACCTGTTAGAGAAGCGG | 1 | 3 |
| PS04 | PS.004 A lp_0340r HvB | CCATCTCATCCCTGCGTGTCTCCGACTCAGccactgGCGTACCTGTTAGAGAAGCGG | Input mix 1-5 |  |
| PS05 | PS.005 A lp_0340r HvB | CCATCTCATCCCTGCGTGTCTCCGACTCAGccagacGCGTACCTGTTAGAGAAGCGG | Input mix 6 |  |
| PS06 | PS.006 A lp_0340r HvB | CCATCTCATCCCTGCGTGTCTCCGACTCAGccagcaGCGTACCTGTTAGAGAAGCGG | 2 | 1.3 |
| PS07 | PS.007 A lp_0340r HvB | CCATCTCATCCCTGCGTGTCTCCGACTCAGccagttGCGTACCTGTTAGAGAAGCGG | 2 | 1.5 |
| PS08 | PS.008 A lp_0340r HvB | CCATCTCATCCCTGCGTGTCTCCGACTCAGccatctGCGTACCTGTTAGAGAAGCGG | 2 | 2 |
| PS09 | PS.009 A lp_0340r HvB | CCATCTCATCCCTGCGTGTCTCCGACTCAGccatggGCGTACCTGTTAGAGAAGCGG | 2 | 3 |
| PS10 | PS.010 A lp_0340r HvB | CCATCTCATCCCTGCGTGTCTCCGACTCAGccattcGCGTACCTGTTAGAGAAGCGG | 2 | 3.2 |
| PS11 | PS.011 A lp_0340r HvB | CCATCTCATCCCTGCGTGTCTCCGACTCAGccgacaGCGTACCTGTTAGAGAAGCGG | Input mix 7 |  |
| PS12 | PS.012 A lp_0340r HvB | CCATCTCATCCCTGCGTGTCTCCGACTCAGccgatcGCGTACCTGTTAGAGAAGCGG | 3 | 1 |
| PS13 | PS.013 A lp_0340r HvB | CCATCTCATCCCTGCGTGTCTCCGACTCAGccgcacGCGTACCTGTTAGAGAAGCGG | 3 | 1.3 |
| PS14 | PS.014A lp_0340r HvB | CCATCTCATCCCTGCGTGTCTCCGACTCAGccgcctGCGTACCTGTTAGAGAAGCGG | 3 | 2 |
| PS15 | PS.015 A lp_0340r HvB | CCATCTCATCCCTGCGTGTCTCCGACTCAGccgcggGCGTACCTGTTAGAGAAGCGG | Input mix 8 |  |
| PS16 | PS.016 A lp_0340r HvB | CCATCTCATCCCTGCGTGTCTCCGACTCAGccgctaGCGTACCTGTTAGAGAAGCGG | Input mix 9 |  |
| PS17 | PS.017 A lp_0340r HvB | CCATCTCATCCCTGCGTGTCTCCGACTCAGccggaaGCGTACCTGTTAGAGAAGCGG | 4 | 0.9 |
| PS18 | PS.018 A lp_0340r HvB | CCATCTCATCCCTGCGTGTCTCCGACTCAGccggccGCGTACCTGTTAGAGAAGCGG | 4 | 1 |
| PS19 | PS.019 A lp_0340r HvB | CCATCTCATCCCTGCGTGTCTCCGACTCAGccgtcgGCGTACCTGTTAGAGAAGCGG | 4 | 2 |
| PS20 | PS.020 A lp_0340r HvB | CCATCTCATCCCTGCGTGTCTCCGACTCAGccgtgtGCGTACCTGTTAGAGAAGCGG | 4 | 3 |
| PS21 | PS.021 A lp_0340r HvB | CCATCTCATCCCTGCGTGTCTCCGACTCAGcctaagGCGTACCTGTTAGAGAAGCGG | 4 | 4 |
| PS22 | PS.022 A lp_0340r HvB | CCATCTCATCCCTGCGTGTCTCCGACTCAGcctaccGCGTACCTGTTAGAGAAGCGG | Input mix 10 |  |
| PS23 | PS.023 A lp_0340r HvB | CCATCTCATCCCTGCGTGTCTCCGACTCAGcctagaGCGTACCTGTTAGAGAAGCGG | 5 | 1.5 |
| PS24 | PS.024 A lp_0340r HvB | CCATCTCATCCCTGCGTGTCTCCGACTCAGcctcgtGCGTACCTGTTAGAGAAGCGG | 5 | 2.3 |
| PS25 | PS.025 A lp_0340r HvB | CCATCTCATCCCTGCGTGTCTCCGACTCAGcctgcgGCGTACCTGTTAGAGAAGCGG | 5 | 3.5 |
| PS26 | PS.026 A lp_0340r HvB | CCATCTCATCCCTGCGTGTCTCCGACTCAGcctgtaGCGTACCTGTTAGAGAAGCGG | Input mix 1-5 with strain WCFS1 100× diluted |  |
| PS27 | PS.027 A lp_0340r HvB | CCATCTCATCCCTGCGTGTCTCCGACTCAGccttatGCGTACCTGTTAGAGAAGCGG | Input mix 1-5 with strain WCFS1 1000× diluted |  |
| PS28 | PS.028 A lp_0340r HvB | CCATCTCATCCCTGCGTGTCTCCGACTCAGccttcaGCGTACCTGTTAGAGAAGCGG | 6 | 1.1 |
| PS29 | PS.029 A lp_0340r HvB | CCATCTCATCCCTGCGTGTCTCCGACTCAGccttgcGCGTACCTGTTAGAGAAGCGG | 6 | 2 |
| PS30 | PS.030 A lp_0340r HvB | CCATCTCATCCCTGCGTGTCTCCGACTCAGcgaacaGCGTACCTGTTAGAGAAGCGG | 1 (scraping from plate) | 1 |
| PS31 | PS.031 A lp_0340r HvB | CCATCTCATCCCTGCGTGTCTCCGACTCAGcgaaggGCGTACCTGTTAGAGAAGCGG | 7 | 1 |
| PS32 | PS.032 A lp_0340r HvB | CCATCTCATCCCTGCGTGTCTCCGACTCAGcgacagGCGTACCTGTTAGAGAAGCGG | 7 | 1.5 |
| PS33 | PS.033 A lp_0340r HvB | CCATCTCATCCCTGCGTGTCTCCGACTCAGcgacgtGCGTACCTGTTAGAGAAGCGG | 7 | 2 |
| PS34 | PS.034 A lp_0340r HvB | CCATCTCATCCCTGCGTGTCTCCGACTCAGcgagcgGCGTACCTGTTAGAGAAGCGG | 7 | 2.5 |
| PS35 | PS.035 A lp_0340r HvB | CCATCTCATCCCTGCGTGTCTCCGACTCAGcgagtaGCGTACCTGTTAGAGAAGCGG | 5 (scraping from plate) | 1.5 |
| PS36 | PS.036 A lp_0340r HvB | CCATCTCATCCCTGCGTGTCTCCGACTCAGcgatccGCGTACCTGTTAGAGAAGCGG | 1 (scraping from plate) | 3 |
| PS37 | PS.037 A lp_0340r HvB | CCATCTCATCCCTGCGTGTCTCCGACTCAGcgatgaGCGTACCTGTTAGAGAAGCGG | 8 | 2 |
| PS38 | PS.038 A lp_0340r HvB | CCATCTCATCCCTGCGTGTCTCCGACTCAGcgattgGCGTACCTGTTAGAGAAGCGG | 8 | 2.5 |
| PS39 | PS.039 A lp_0340r HvB | CCATCTCATCCCTGCGTGTCTCCGACTCAGcgcactGCGTACCTGTTAGAGAAGCGG | 8 | 3.2 |
| PS40 | PS.040 A lp_0340r HvB | CCATCTCATCCCTGCGTGTCTCCGACTCAGcgcatgGCGTACCTGTTAGAGAAGCGG | 8 | 3.5 |
| PS41 | PS.041 A lp_0340r HvB | CCATCTCATCCCTGCGTGTCTCCGACTCAGcgccacGCGTACCTGTTAGAGAAGCGG | 8 | 4 |
| PS42 | PS.042 A lp_0340r HvB | CCATCTCATCCCTGCGTGTCTCCGACTCAGcgcgatGCGTACCTGTTAGAGAAGCGG | 8 | 4.5 |
| PS43 | PS.043 A lp_0340r HvB | CCATCTCATCCCTGCGTGTCTCCGACTCAGcgcggaGCGTACCTGTTAGAGAAGCGG | 9 | 0.15 |
| PS44 | PS.044 A lp_0340r HvB | CCATCTCATCCCTGCGTGTCTCCGACTCAGcgcgtcGCGTACCTGTTAGAGAAGCGG | 9 | 1.5 |
| PS45 | PS.045 A lp_0340r HvB | CCATCTCATCCCTGCGTGTCTCCGACTCAGcgctagGCGTACCTGTTAGAGAAGCGG | 9 | 2 |
| PS46 | PS.046 A lp_0340r HvB | CCATCTCATCCCTGCGTGTCTCCGACTCAGcgcttaGCGTACCTGTTAGAGAAGCGG | 9 | 3 |
| PS47 | PS.047 A lp_0340r HvB | CCATCTCATCCCTGCGTGTCTCCGACTCAGcggaagGCGTACCTGTTAGAGAAGCGG | 9 | 3.5 |
| PS48 | PS.048 A lp_0340r HvB | CCATCTCATCCCTGCGTGTCTCCGACTCAGcggaccGCGTACCTGTTAGAGAAGCGG | 1 (scraping from plate) | 2 |
| PS49 | PS.049 A lp_0340r HvB | CCATCTCATCCCTGCGTGTCTCCGACTCAGcggagtGCGTACCTGTTAGAGAAGCGG | 10 | 2 |
| PS50 | PS.050 A lp_0340r HvB | CCATCTCATCCCTGCGTGTCTCCGACTCAGcggataGCGTACCTGTTAGAGAAGCGG | Input mix 1-5 with strain WCFS1 10000× diluted |  |
| PS51 | PS.051 A lp_0340r HvB | CCATCTCATCCCTGCGTGTCTCCGACTCAGcggcaaGCGTACCTGTTAGAGAAGCGG | 5 (scraping from plate) | 2.3 |
| Q1 | Lp-16Sfo(2)^e^ | TGATCCTGGCTCAGGACGAA | Total *L. plantarum* population |  |
| Q2 | Lp-16Sre(2)^e^ | TGCAAGCACCAATCAATACCA | Total *L. plantarum* population |  |
| Q3 | Q-PCR_10LP_strains_F | GCGGGTGGCGAAGGCTATGTGCGC | 339-IR-340 |  |
| Q4 | Q-PCR_10LP_strains_R | CGAATAAGTGCAGTTTTGCAATTCGC | 339-IR-340 |  |

^a^ Primers starting with PS in the name are the primers used for pyrosequencing.

^b^ Nucleotides in non-capitals are the barcode and underlined nucleotides are complementary to the *L. plantarum* strain DNA.

^c^ If applicable.

^d^ All primers starting with PS in the name are combined with this forward primer.

^e^ Reference for primers Q1 and Q2 is . The other primers were designed in this work.

**References**

1. Bron PA, Marco M, Hoffer SM, Van Mullekom E, de Vos WM, et al. (2004) Genetic characterization of the bile salt response in *Lactobacillus plantarum* and analysis of responsive promoters *in vitro* and *in situ* in the gastrointestinal tract. J Bacteriol 186: 7829-7835.
