# Supplementary material for: Congruent Strain Specific Intestinal Persistence of Lactobacillus plantarum in an Intestine-Mimicking In Vitro System and in Human Volunteers
Source: PLoS One. 2012 Sep 6;7(9):e44588. doi: 10.1371/journal.pone.0044588 (PMC3435264; doi:10.1371/journal.pone.0044588)
Supplement: Table S3 — Primer pair combinations used for intergenic variable region amplification and summary of the subsequent PCR and sequencing results. (DOC) [file pone.0044588.s005.doc]

Table S3. **Primer pair combinations used for intergenic variable region amplification and summary of the subsequent PCR and sequencing results.**

| **Primer combinations** | **Expected product length (bp)** | **PCR and sequencing results including observed products lengths (bp)** | **Further use** |
| --- | --- | --- | --- |
| A + B | 530 | Seven out of 8 strains yielded a weak product at 530 + non-specific products | No |
| C + D | 800 | All 8 tested strains yielded a product at 800 + non-specific products | Yes |
| E + F | 420 | No products | No |
| G + H | 400 | Six out of 8 strains yielded a product at 400 + non-reliable sequence results | No |
| I + J | 360 | All 8 tested strains yielded a product at 360, but little variation was observed | No |
| K + L | 600 | Four out of 8 strains yielded a product at 600, possible prophage | No |
| M + N | 690 | Thirteen out of 19 strains yielded a product at 690 + variation observed, possible prophage | No |
| O + P | 450 | Four out of 12 stains yielded a product at 450 | No |
| Q + R | 460 | Six out of 8 strains yielded a product at 460 + 2 strains yielded non-specific products | No |
| S + T | 360 | One out of 8 strains yielded a product at 360 | No |
| U + V | 500 | All 19 strains yielded a product at 500, but little sequence variation was observed | No |
